# Primary solitary pituitary stalk abscess: case report and review

**DOI:** 10.3389/fendo.2025.1510593

**Published:** 2025-04-01

**Authors:** Yuehui Ma, Linghao Bu, Jing Jin

**Affiliations:** Department of Neurosurgery, the First Affiliated Hospital, School of Medicine, Zhejiang University, Hangzhou, China

**Keywords:** pituitary stalk, abscess, primary, diagnosis, surgery

## Abstract

**Introduction:**

Pituitary stalk (PS) abscess has not been previously reported. In this study, we report a case of PS abscess presenting with central diabetes insipidus (CDI), hypopituitarism, hyperprolactinemia, and blurred vision. We highlight radiological clues and pathological biopsy findings to clarify the diagnosis and review the literature.

**Case presentation:**

A 67-year-old female presented with a > 3-month history of bitter and dry mouth, polyuria, blurred vision, fatigue, and poor appetite without inducement. Laboratory investigations revealed CDI, pituitary-thyroid, and pituitary-gonadal axis hypofunction, decreased insulin-like growth factor-1, and slightly elevated prolactin levels. Magnetic resonance imaging (MRI) revealed an isolated cystic, thickened PS with ring enhancement. The patient underwent transsphenoidal surgery. Direct observation during surgery revealed a PS abscess and pale-yellow pus. Histopathological evaluation showed PS tissue with inflammatory cell invasion and lymphocyte proliferation. The patient was treated with linezolid and ceftriaxone for 4 weeks post-surgery and levothyroxine, hydrocortisone, and desmopressin replacement therapy. MRI showed no signs of recurrence of the PS abscess 3 years post-surgery.

**Conclusion:**

This case reports a newly identified solitary PS lesions characterized by cystic PS thickening and ring enhancement on MRI, presenting with CDI, hyperprolactinemia, hypopituitarism, and blurred vision. The patient recovered uneventfully, and the postoperative MRI was normal without any recurrent lesions.

## 1 Introduction

Pituitary stalk (PS) lesions are rare and typically identified on magnetic resonance imaging (MRI) during investigations for central diabetes insipidus (CDI) and/or hypopituitarism. The pathological etiologies of PS lesions are complex and diverse and usually fall into three categories: neoplastic, inflammatory and infectious, and congenital and developmental (1–3). Due to their often subtle clinical and radiographic presentations, PS lesions present a significant diagnostic challenge for clinicians. PS lesions may arise from the PS itself or extend from pathological processes involving the pituitary gland and/or hypothalamus. The most common inflammatory and infectious diseases associated with isolated PS lesions are lymphocytic hypophysitis and Langerhans cell histiocytosis (4). PS infections are rare and mostly described in case reports or small case series, typically resulting from hypothalamic-pituitary infections (5–7).

In this study, we report an extremely rare case of a solitary PS abscess, highlight clinical and radiological clues and pathological biopsy findings to clarify the diagnosis, and review the literature.

## 2 Case presentation

A 67-year-old female presented with a > 3-month history of bitter and dry mouth, polyuria, blurred vision, fatigue, and poor appetite without inducement. The patient’s fasting blood glucose was 4.59 mmol/l, hemoglobin A1 6.1%, hemoglobin A1c 5.1%, and the blood glucose at 1, 2, and 3 hours after the meal was 9.73 mmol/l, 7.85 mmol/l, and 5.67 mmol/l, respectively. The patient had no symptoms of fever, headache, vision rotation, or dizziness and was instructed to improve their lifestyle and dietary habits to adjust her blood sugar levels. However, the patient’s condition did not improve. She had been in good physical condition and denied a history of diabetes mellitus, tuberculosis and immunocompromised/human immunodeficiency virus (HIV), but had a 10-year history of untreated thyroid nodules and hypertension.

The patient was admitted to the endocrinology department of our hospital in August 2021. On examination, she was slightly listless, with a blood pressure of 135/75 mmHg. A partial binocular temporal field defect was observed on perimetry; however, no other neurological deficits were observed. Laboratory investigations, including complete blood count, erythrocyte sedimentation rate, C-reactive protein (CRP) levels, serum antibodies of mycobacterium tuberculosis, syphilis and HIV and tumor-related markers, were all within the normal range, except for a slight increase in CRP (10.1 mg/L). Evaluation of the pituitary hormone profile showed evidence of hypopituitarism (**Table 1**). However, the patient’s serum prolactin (PRL) was slightly elevated (36.42 ng/mL). Urinalysis indicated a urine-specific gravity of 1.000 with no signs of infection. Biochemical profile revealed hypernatremia (151 mmol/L), and serum and urine osmolarity was 296 mmol/kg and 53 mmol/kg, respectively. Water deprivation and desmopressin testing confirmed a diagnosis of complete CDI.

**Table 1:** The endocrinology test before surgery and during follow-up.

Test	Before surgery	One year after surgery	Reference value
TT3 (nmol/L)	0.77	1.44	0.98–2.33
FT3 (pmol/L)	2.13	3.14	2.43–6.01
TT4 (nmol/L)	60.97	89.46	62.68–150.84
FT4 (pmol/L)	8.06	9.87	9.01–19.05
TSH (mIU/L)	0.859	1.311	0.350–4.940
ACTH (pg/mL,8 AM)	11.6	17.41	0.00–46.00
Cortisol (μg/dL,8 AM)	15.2	23.9	5.00–25.00
ACTH (pg/mL,4 PM)	7.89	7.56	0.00–46.00
Cortisol (μg/dL,4 PM)	14.00	14.7	5.00–25.00
Cortisol (μg/dL,0 AM)	7.78	–	–
24h-UFC (μg/24h)	85.9	128.7	20.9–292.3
GH (ng/mL)	0.13	0.82	0.00–8.00
IGF-1 (ng/mL)	56.6	3.8	69.0–200.0
IGFBP-3 (ug/mL)	2.1	80.8	3.0–6.2
FSH (mIU/mL)	8.59	14.60	Follicular phase:3.03–8.08, Middle menstrual period:2.55–16.69, Luteal phase:1.38–5.47, Postmenopause:26.72–133.41
LH (mIU/mL)	2.18	3.16	Follicular phase:1.80–11.78, Middle menstrual period:7.59–89.08, Luteal phase:0.56–14.00, Postmenopause:5.16–61.99
PRL (ng/mL)	36.42	11.92	5.18–26.53

TT3, total triiodothyronine; FT3, free triiodothyronine; TT4, thyroxine; FT4, free thyroxine; TSH, thyroid-stimulating hormone; ACTH, adrenocorticotropic hormone; UFC, urinary free cortisol; GH, growth hormone; IGF-1, insulin-like growth factor-1; IGFBP-3, insulin-like growth factor-binding protein-3; LH, luteinizing hormone; FSH, follicle-stimulating hormone; PRL, prolactin.

**Figure 1:**
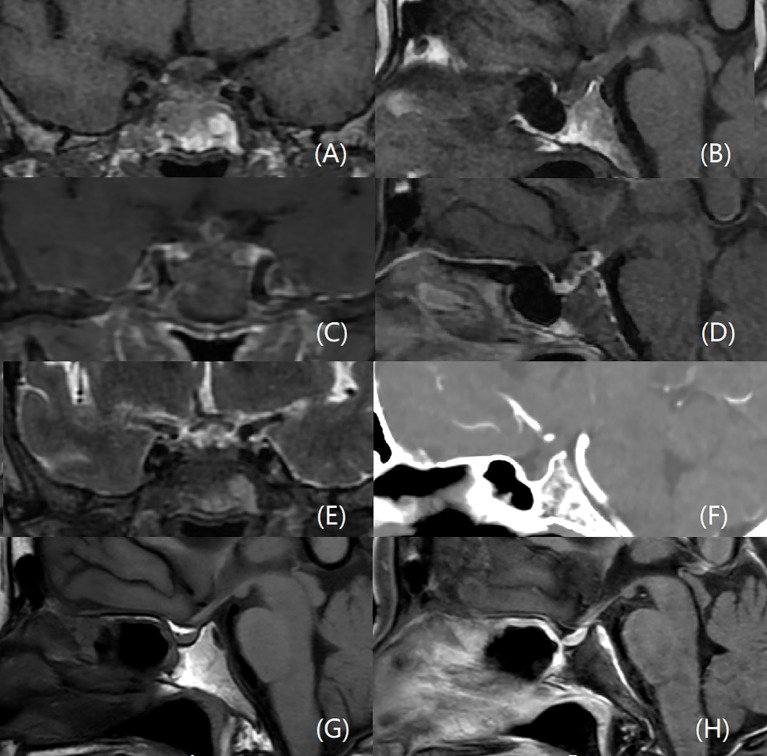
MRI and CT scans of the PS abscess before and after surgery. An enlarged PS with heterogeneous iso-hypointense on coronal **(A)** and sagittal **(B)** T1W, and iso-hyperintense signals on coronal T2W images **(E)**. The lesion is ring-enhancing after gadolinium administration on coronal **(C)** and sagittal **(D)** T1W images. There is no bright spot of posterior pituitary **(B)**. There are no other lesions elsewhere in the brain, and no meningeal enhancement. CT scan reveals a thickening and isodensity PS **(F)**. The PS is normal three years after surgery, **(G, H)** represent sagittal sections of the PS before and after contrast administration, respectively.

Due to the presence of polyuria and visual field defects, the patient underwent dynamic enhanced MRI of the pituitary gland, which showed a thickened PS with heterogeneous iso-hypointense and iso-hyperintense signals on T1W and T2W images, respectively, indicating a cystic lesion (**Figure 1**). After gadolinium administration, the lesion exhibited ring enhancement on T1W images. There were no other lesions elsewhere in the brain, and there was no meningeal enhancement to suggest meningitis. Cranial computed tomography (CT) revealed PS thickening and isodensity. Given the PS imaging abnormalities, a lumbar puncture was performed; however, cerebrospinal fluid analysis showed no biochemical or cytological abnormalities.

**Figure 2:**
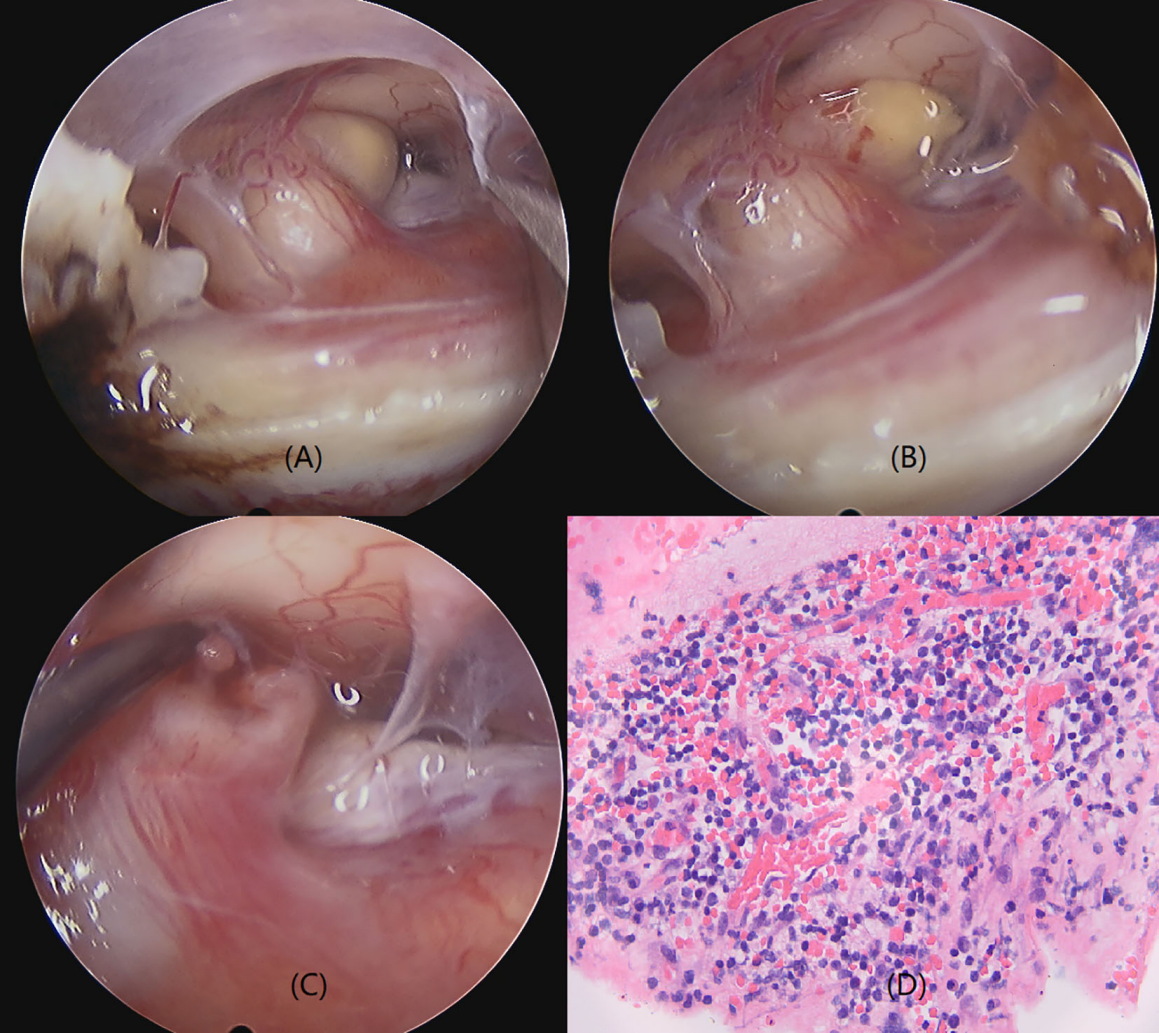
Operative view and pathology of the PS abscess. PS is thickened and pale yellow with a yellowish cystic protrusion and yellow viscous fluid on the left side of the PS **(A, B)**. A small nodule is observed in the cyst cavity of the PS **(C)**. PS thickening improved, and the color of the PS returned to pink after the fluid was cleaned up. The pathology of the nodule demonstrates a pituitary stalk tissue with dense infiltration of inflammatory cells and proliferation of lymphocytes **(D)** (Hematoxylin eosin staining, 400×).

The patient had panhypopituitarism secondary to a PS lesion of unclear pathological origin. Levothyroxine, hydrocortisone, and desmopressin replacement therapy was initiated, and the patient was referred for an endoscopic transsphenoidal biopsy. Direct observation during surgery revealed that the PS was thick and pale yellow (**Figure 2**). A yellowish cystic protrusion was observed on the left side of the PS. When the cyst was cut open, a yellow viscous fluid gushed out. However, these fluids were not obtained. After the fluid was cleaned, the thickening of the PS improved, and the color of the PS gradually returned to pink. Except for a small nodule in the cystic cavity of the PS, no other abnormal hyperplastic tissues were observed. Therefore, we used nodule tissue for pathological examination. Histopathologic analysis revealed PS tissue with dense inflammatory cell infiltration and lymphocyte proliferation; however, no atypical cells were observed. The final diagnosis was a PS abscess (**Figure 2**).

Postoperatively, the patient was treated with linezolid (600 mg, twice daily) and ceftriaxone (2 g, twice daily). When pathology was confirmed, the above-mentioned regimen was maintained for 4 weeks. The patient recovered uneventfully and was discharged on levothyroxine (50 ug/d), hydrocortisone (30 mg/d), and desmopressin (0.2 mg/d) replacement therapy. The blurred vision of the patient was restored three months after operation. One year later, endocrine laboratory results showed improvement in all indicators (**Table 1**). Owing to improvement in CDI, replacement therapy (desmopressin, levothyroxine, and hydrocortisone) was discontinued. The patient has been followed for 3 years, with repeat MRI showing a normal-sized PS and normal MRI (**Figure 1**).

Although the patient initially required medication for diabetes insipidus post-surgery, her condition gradually improved, allowing for the eventual discontinuation of all drugs. The patient expressed satisfaction with the treatment and outcomes.

## 3 Discussion

We report a rare case of a PS abscess in a female who presented with CDI, bitter mouth, blurred vision, fatigue, and poor appetite. According to our literature search, no similar cases have been documented to date. Due to the critical location and function of the PS, clinicians are often hesitant to perform biopsies because of concerns over iatrogenic hypopituitarism. Usually, the etiological diagnosis of PS lesions relies on clinical evaluation and imaging findings. This case provides updated clinical and radiological information that may assist clinicians when encountering similar PS lesions.

Patients with PS pathology often present with varying degrees of CDI, hypopituitarism, and hyperprolactinemia (3, 4, 8). Our patient had no history or recent indications of systemic or intracranial infection. This aligns with reports on pituitary abscesses, where symptoms like high fever and frank hyper-leukocytosis are rare (9). Therefore, the patient’s clinical presentation was nonspecific and similar to that of other PS lesions. CDI has been reported as the most common endocrine abnormality in PS lesions. There was probably loss of bright spot of posterior pituitary responsible for diabetes insipidus symptoms of polyuria. Studies on the incidence of adenohypopituitarism have reported contradictory results. Turcu et al. (3) found that secondary hypogonadism was the most common, whereas adrenal insufficiency was the least common. Yoon et al. (8) reported that secondary hypothyroidism was the most common, followed by low levels of insulin-like growth factor-1 or growth hormone deficiency, whereas hypogonadism was the least common. Therefore, hypopituitarism in PS lesions was not associated with the disease.

MRI of the hypothalamus and pituitary region is extremely useful for investigating PS lesions, especially in conjunction with the clinical context. The MRI features of PS lesions include PS thickening, abnormal signal intensities, and various enhancement patterns. However, MRI characteristics of PS inflammatory lesions have not been shown to correlate specifically with a particular disease, except in cases of PS neurosarcoidosis, which typically presents with uniform thickening and enhancement confirmed by pathology (3). Isolated PS enlargement is commonly seen in lymphocytic hypophysitis, Langerhans cell histiocytosis, and metastatic tumors too (4). Both neurosarcoidosis and lymphocytic hypophysitis would cause homogenous enhancement, however abscess would have peripheral enhancement. In our case, the isolated and thickened PS displayed MRI signals consistent with a cystic lesion and ring enhancement, similar to a brain abscess. Although this cystic feature of a PS lesion had not been previously reported, we believe it may be a characteristic signal of a PS abscess. Although very rare, Rathke’s cleft cyst infection and tuberculosis should also be considered in the differential diagnosis. Turcu et al. reported a group of 92 cases of PS lesions, 2 of which were Rathke’s cleft cyst, which showed a nonenhancing, well-demarcated and round/diamond enlargement of PA on MRI (3). However, Rathke’s cleft cyst infections was only reported in the pituitary area, which showed a cystic sellar with T1 hyperintense and T2 hypointense. There was increased enhancement of the adenohypophysis due to hyperemia (15). These were significantly different from the imaging findings of our case. Tuberculosis can affect the pituitary and PA via the presence of a tuberculoma (2, 16). Only rare cases of isolated tuberculoma of the PA have been reported (17). Tuberculoma was often a strongly enhancing mass with T1 isointense-to-hypointense and T2 hyperintense, or may appeared as a hyperintense center surrounded by a hypointense rim with peripheral ring enhancement. Imaging could also reveal involvement of the paranasal sinuses or pituitary fossa, and adjacent meningeal enhancement (6, 18, 19). Given the absence of any history of infection or MRI evidence of meningoencephalitis, it was also essential to differentiate the diagnosis from craniopharyngioma and metastatic tumors.

Pituitary abscesses are rare and life-threatening (9). We believe that PS abscesses are similar to pituitary abscesses. In this case, the PS abscess was the primary lesion, with no preexisting sellar lesions. Endoscopic transsphenoidal drainage may be the most effective and safe approach for patients with PS abscesses. In this case, pus was not obtained for microbiological evaluation or culture. Due to the inconclusiveness of the pathology, the microbial etiology of PS abscess remains unclear, and no similar cases have been reported in the literature for reference. Microbial agents in pituitary abscesses are usually gram-positive pathogens, such as Staphylococcus aureus and various Streptococcus species. Gram-negative bacteria, including Escherichia coli and Pseudomonas aeruginosa, and fungal agents are found less frequently (9–11). The medical treatment in our case was based on literature reports on the common microbial infection of pituitary abscesses and was treated with linezolid and ceftriaxone (11–13). The therapeutic outcomes in our case were good. Hypopituitarism recovered to some extent. There was no recurrence of the PS abscess 3 years post-surgery, consistent with the fact that postoperative recurrence of a pituitary abscess is uncommon (12, 14).

## 4 Conclusions

In conclusion, this case report describes a new type of PS lesion characterized by cystic PS thickening and ring enhancement on MRI, accompanied by CDI, hyperprolactinemia, hypopituitarism, and blurred vision. When these conditions occur in PS lesions, a PS abscess should be considered in the differential diagnosis. Systemic evaluation of clinical, hormonal, and radiological findings is essential for accurate diagnosis and differentiation from neoplastic and other inflammatory lesions.

## Data Availability

The original contributions presented in the study are included in the article/supplementary material. Further inquiries can be directed to the corresponding author.
